# Older adults’ lower-limb muscle power production throughout a full flight of stairs: Reliability and comparison between different stair models

**DOI:** 10.1371/journal.pone.0296074

**Published:** 2024-02-15

**Authors:** Lien Meulemans, Evelien Van Roie, Jan Seghers, Christophe Delecluse

**Affiliations:** Department of Movement Sciences, Physical Activity, Sports & Health Research Group, KU Leuven, Leuven, Belgium; Brunel University London, UNITED KINGDOM

## Abstract

Lower-limb muscle power should be closely monitored to prevent age-related functional ability declines. Stair-climbing (SC) power is a functionally relevant measurement of lower-limb muscle power. Body-fixed sensors can measure power production throughout the different steps of a flight of stairs to assess different aspects of performance. This study investigated: 1) power production throughout a full flight of stairs; 2) if staircases with less or more steps can provide similar information; and 3) test-retest reliability of SC power. 116 community-dwelling older adults (57 women) ascended three staircases as fast as possible: 12, 6 and 3 steps. Mean vertical power production per step was collected and analyzed using a commercial body-fixed sensor and software. Three phases were found in SC power production: 1) an acceleration phase, i.e., the power produced in step 1 (P_1_); 2) a phase where the highest performance (P_max_) is reached and; 3) a fatiguing phase with power loss (P_loss_; only measurable on 12-step staircase). Mean power (P_mean_) over the different steps was also evaluated. P_1_ did not differ between staircases (all p>0.05), whereas P_max_ and P_mean_ were higher with increasing number of steps (p = 0.073 –p<0.001). P_1_, P_max_ and P_mean_ were strongly correlated between staircases (r = 0.71–0.95, p<0.05). and showed good to excellent reliability (ICC = 0.66–0.95, p<0.05). P_loss_ showed poor reliability. To conclude, measurements of SC power production (P_1_, P_max_ and P_mean_) with a single sensor on the lower back are reliable across different staircases. A small, transportable, 3-step staircase can be used for measuring power production in clinical practices with no access to regular staircases. However, absolute values are dependent on the number of steps, indicating that measurements to track performance changes over time should always be done using an identical stair model.

## Introduction

Aging is associated with declines in lower-limb muscle strength and power [1–3]. Muscle power (i.e., force x velocity) starts to decline earlier in life and at a faster rate compared to muscle mass and strength [4–6]. Low values of muscle power in old age have been associated with mobility limitations [7, 8] and mortality [9, 10], stressing the importance to include muscle power assessments to prevent these negative outcomes.

Golden standard methodologies for measuring lower-limb muscle power (e.g. a leg press device) are quite complex, which limits applicability in daily life [5, 11]. Clinically feasible measurements of lower-limb muscle power are needed for implementing muscle power assessments in daily-life practice [12]. Lower-limb muscle power can be measured during activities of daily living, like standing up from a chair [13] or stair climbing (SC) [14, 15]. The ability to climb stairs is essential for independent living in community environments [16, 17]. Furthermore, SC can be considered as a safe and feasible test in community-dwelling older adults [18]. Bean and his colleagues (2007) used the so called ‘SC power test’ or SCPT, which is an estimation of mean SC power based on the time needed to ascent the stairs (measured with a stopwatch), the vertical height of the stair and the subject’s body mass. The SCPT on a 10-step staircase was compared with power production on a leg press device, showing a correlation of 0.52 [14]. However, large staircases are not often available in clinical settings. For this reason, Ni and colleagues (2017) developed a 4-step stair model. Results showed excellent reliability (ICC = 0.95) and strong criterion validity (r = 0.85–0.86) of the 4-step SCPT [19] compared to a leg press device in a sample of 50 community-dwelling older men and women.

Although the SCPT is easy to use and enables frequent muscle power measurements, the mathematic equation only estimates a mean power value across the full flight of stairs [14]. In reality, power production can differ from step to step in stair climbing. The lack of kinematic data does not allow measuring power fluctuations throughout a flight of stairs, which can give valuable information on different aspects of performance, e.g. initial acceleration, maximum performance, fatigue-related drop in performance.

Kinematic data can be collected by means of body-fixed sensors, providing data on power production during every step of a stair [15, 20]. We have previously demonstrated that sensor-based SC power is highly related (r = 0.80) to leg-extensor power in a group of young, middle-aged and older adults [15]. To the author’s knowledge, no research has investigated power production throughout the different steps on a regular staircase of 10–15 steps. It is also unknown whether staircase models with a smaller number of steps can provide similar information as a regular staircase. This is important to verify, considering that many clinical settings have limited space or no access to a full flight of stairs. Therefore, the current study aimed at: 1) investigating power production per step throughout a full flight of stairs by using a body-fixed sensor and defining parameters of interest; 2) comparing SC performance on different stair models in community-dwelling older adults; and 3) evaluating the test-retest reliability of power parameters during SC.

## Materials and methods

### Subjects and study design

Community-dwelling men and women aged 65 years and older were recruited via local advertisements to participate in a cross-sectional study on stair-climbing performance. The following exclusion criteria were applied: unable to climb stairs, unstable cardiovascular disease, dementia, recent surgery, infection and musculoskeletal injury. Our aim was to recruit a minimum of N = 100 (50 per sex) subjects. In total, 116 subjects agreed to participate in the study. Sensitivity analyses in G*Power indicated that a sample of N = 116 is able to detect a small effect size (f = 0.11) in a repeated measures ANOVA (power = 0.95; α 0.05; 2 groups (sex); 3 measurements (3-6-12 step stair)). To assess the test-retest reliability of stair-climbing parameters, 31 men and 22 women participated in a retest within 2 to 6 days after the initial test session. This sample size for test-retest reliability was based on Hopkins et al. [21], indicating that at least 50 subjects is recommended. Both sessions occurred in the same lab and under guidance of the same researcher. The study was approved by the Ethics Committee Research UZ/KU Leuven in accordance with the Declaration of Helsinki (S62540). All subjects provided a written informed consent. Measurements were performed from January 2020 until March 2022.

### Anthropometric and demographic measurements

Body weight (kg) and height (m) were determined with a basic scale device and a standard stadiometer. Body mass index (BMI; kg/m^2^) was calculated with following formula: weight/(height^2^). Education level and presence of chronic conditions were collected by means of a questionnaire. Functional status was determined with the Short Physical Performance Battery (SPPB), as described elsewhere [22].

### Stair-climbing test

**Fig 1:**
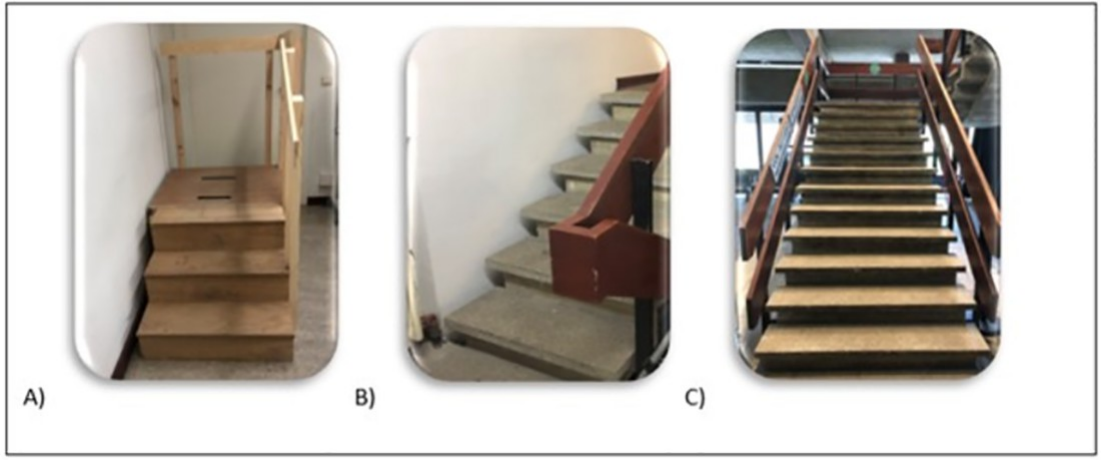
An overview of the three different staircases. A) a 3-step stair model, newly developed for this study (wooden construction with the following dimensions: Height = 18cm, Width = 70cm, Depth = 25cm (including 2cm nosing) for each step; Height = 54cm, Width = 80cm, Depth = 80cm for the platform on top); B) a regular staircase with 6 steps (a concrete staircase with the following dimensions for each step: Height = 18cm, Width = 70cm, Depth: 26cm (including 4.5cm nosing)) and; C) a reference staircase of 12 steps (a concrete staircase with the following dimensions for each step: Height = 17cm, Width = 150cm, Depth: 29cm (including 5cm nosing)).

Subjects were instructed to ascend three different stair models as fast as possible without using the handrail and without skipping a step: a reference staircase of 12 steps, a shorter staircase of 6 steps and a newly developed 3-step stair model (Fig 1). To ensure maximal performance, subjects were asked not to stop abruptly at the top of the stairs but to take two extra steps before standing still. Step dimensions were similar across the different stairs (18 cm height). After a familiarization trial, subjects performed three trials (30-60s rest between trials) of each stair model and the order of the stair models was randomized. Two to five minutes of rest were provided in between conditions. During the test, subjects wore an elastic belt around their waist that positioned a sensor (DynaPort MoveTest, McRoberts, The Hague, NL) on the middle of the lower back. The sensor included a tri-axial accelerometer and gyroscope and was positioned so that it was close to the body’s center of mass. The sensor had a sampling rate of 100 Hz and commercially available software (DynaPort MoveTest, McRoberts, The Hague, NL) was used to analyze the data. Every single step during stair ascent was analyzed. A step was divided in two subphases based on the vertical displacement: a rise phase, where there is vertical displacement, and a support or stance phase, where there is no vertical displacement. A more detailed description of this methodology can be found elsewhere [15]. Total ascent duration (s) was calculated as the sum of the durations for all steps of each stair model. Instantaneous power (W) was calculated as body mass x (vertical acceleration + 9.81m/s^2^) x vertical velocity x cos (angle between the vertical velocity and vertical force vector). Mean power values (P, in W) were calculated for each single rise phase according to previous procedures [15]. All power values were divided by the individual’s body mass to obtain relative power values (W/kg). Only the best trial of each stair model, based on lowest total duration, was included in the analyses.

To note, we first explored the data on mean power production throughout the different steps of a flight of stairs (see results section). Based on this exploration, parameters of interest were defined for the remaining statistical analyses. These parameters are described in the results section.

### Statistical analysis

Statistical analyses were performed with SPSS (version 28.0.1.1) and Rstudio (version 1.4.1564). Level of significance was set at p < 0.05. To examine stair climbing power production throughout a flight of stairs, relative mean power values were plotted for every single step. The 12-step staircase was identified as the reference stair, given that–in most houses–staircases have about 12 steps to move from one floor to another. Standard descriptive statistics (means ± SD’s) were used to describe the data.

To examine differences on power and duration parameters between the three stair models (3-6-12 steps) and between sex, linear mixed models were built using the function lmer provided by the R-package lme4. Stair model and sex were implemented as fixed effects in the regression models (model 1). If there was a main effect of stair model and sex, a second model was built by including the interaction term ‘stair model-by-sex’ as fixed effect in the regression model (model 2). Subject was included in the models as random effect to correct for the repeated measures design.

To examine the relationship between the three different stairs, power and duration parameters were compared with Pearson’s correlation coefficients (Pearson’s r) (separately for men and women). Pearson’s r can be interpreted as <0.39 weak, 0.40–0.69 moderate, 0.70–0.89 strong and 0.90–1.00 very strong correlation [23].

For the reliability analyses of the subsample, mean differences and SD’s were calculated. Relative reliability was determined using intraclass correlation coefficients (ICC_3,1_) and their 95% confidence intervals (95% CI). ICC’s were interpreted as <0.50 poor, 0.50–0.75 moderate, 0.75–0.90 good and >0.90 excellent reliability [24]. Absolute reliability was determined using the coefficient of variation (CV) and the minimal detectable change (MDC), calculated via following formulas: CV (%) = 100 x [2x (SDdifference / √2)/(Mean1 + Mean2)] and MDC = SEM x 1.96 x √2 (with SEM calculated as the square root of the residual mean square error from the repeated measures analysis of variance). MDC values were expressed as a percentage of the mean (MDC%) to make easy comparisons possible.

## Results

Descriptives of the study sample can be found in Table 1.

**Table 1:** Descriptive overview of the study sample (n = 116; ♂59 ♀57).

Variable		Mean ± SD
Age (years)		70.89 ± 5.97
Weight (kg)		74.91 ± 13.92
Height (m)		1.68 ± 0.09
BMI (kg/m^2^)		26.39 ± 3.85
SPPB score		11.82 ± 0.55
Educational level^a^ (n)	Bachelor’s or master’ degree	69
	Upper secondary education	35
	Lower secondary education	7
	Primary education	3
Number of chronic conditions^a^ (n)	0	68
	1	31
	2	11
	3	3
	4	1

BMI = body mass index, SPPB = short physical performance battery

^a^Questionnaire data of 2 subjects are missing. Chronic conditions were defined as: hypercholesterolemia, hypertension, diabetes, (rheumatoid) arthritis, thyroid problems, gastro-intestinal problems, cardiovascular disease, kidney disease, depression, musculoskeletal problems, chronic pain, cancer, osteoporosis, fibromyalgia, skin conditions and neurological problems.

### Description of power production throughout a flight of stairs

**Fig 2:**
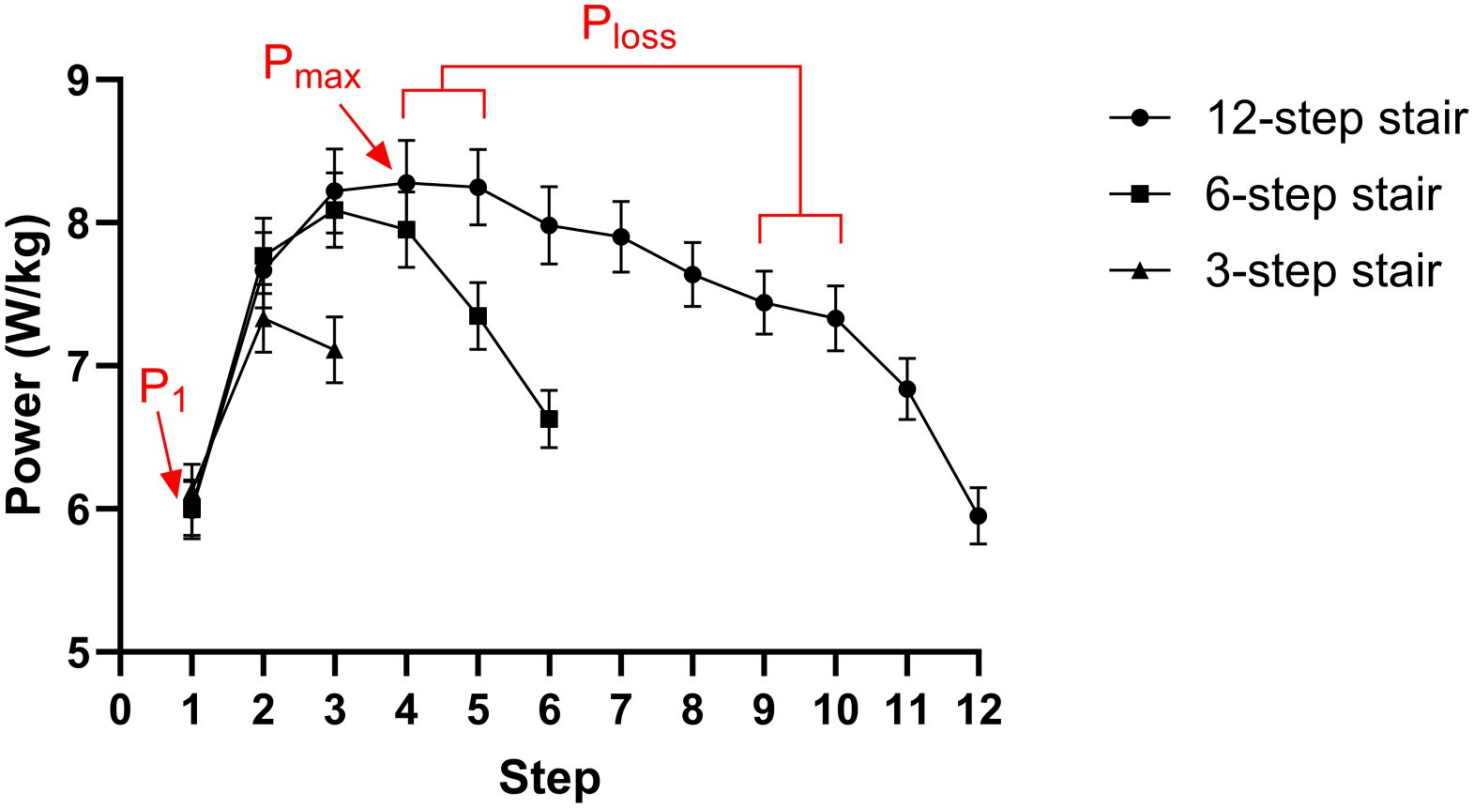
Stair-climbing (SC) power per step on a 3-, 6- and 12-step stair model. Dots and bars represent means and SEM’s, respectively. The three different phases of SC (i.e., P_1_, P_max_ and P_loss_) are indicated for the 12-step stair. P_1_ = power in step 1; P_max_ = highest power production; P_loss_ = loss in power.

Fig 2 gives an overview of stair climbing performance, i.e. relative mean power per step, on the three different stairs. The drop in performance in the final two steps of the 6-step model (i.e., step 5 and 6) compared to step 5 and 6 of the 12-step model (both p<0.001) indicates that the performance is negatively influenced by a breaking phase near the end of the stair (and not by fatigue). The subjects seem to anticipate on the end of the stair, resulting in a reduction in power at step 5 and 6 in the 6-step model, which is not visible in the 12-step model. Here, a similar deceleration was found in the last two steps (i.e., 11 and 12). Likewise, the last step on the 3-step model (i.e., step 3) is significantly lower compared to the corresponding step on the 6- and 12-step staircase (p<0.001). This indicates that the final step (3-step model) or the final two steps (6- and 12-step model) of a stair test should not be used as an indicator of performance.

The reference stair of 12 steps enables us to distinguish between three phases in SC performance: 1) an acceleration phase, where power is build up after the start of the movement in step 1 (P_1_); 2) a phase where the highest performance (P_max_) is reached and; 3) a fatiguing phase, where there is a loss in power (P_loss_), here calculated as the percentual difference between the relative mean power on step 4–5 and the relative mean power of step 9–10. To note, for the calculation of P_loss_ two steps were averaged so that left-right differences would not influence the calculation of the parameter. In the 3- and 6-step stair model, only P_1_ and P_max_ can be estimated.

### Comparison between different stair models

Descriptives of P_1_, P_max_ and P_loss_ can be found in Table 2 for men and women separately. We additionally report the overall mean power over the different steps (P_mean_), as this is the only parameter that can be estimated based on total duration (as done in previous work with estimation formulas [14]).

**Table 2:** Descriptive overview of stair-climbing parameters and results of linear mixed model analyses investigating differences between stair models (3-6-12 step) and between men (♂, n = 59) and women (♀, n = 57).

	3-step stair		6-step stair		12-step stair		♂ vs ♀	3 vs 6	3 vs 12	6 vs 12
	♂	♀	♂	♀	♂	♀	p-value	p-value	p-value	p-value
P_1_ (W/kg)	6.87 ± 2.11	5.36 ± 1.52	6.81 ± 2.30	5.17 ± 1.52	6.77 ± 2.30	5.18 ± 1.69	<0.001	0.327	0.261	0.885
P_max_ (W/kg)	8.92 ± 2.43	6.62 ± 1.99	10.19 ± 2.68	7.20 ± 2.20	10.90 ± 2.95	7.45 ± 2.30	<0.001	<0.001	<0.001	0.073
P_loss_ (%)	/	/	/	/	-9.95 ± 13.57	-7.45 ± 11.59	0.289^a^	/	/	/
P_mean_ (W/kg)	7.81 ± 2.13	5.86 ± 1.76	8.38 ± 2.24	6.18 ± 1.94	8.75 ± 2.24	6.15 ± 1.90	<0.001	<0.001	<0.001	0.054
Total duration (s)	0.86 ± 0.18	0.99 ± 0.21	1.75 ± 0.31	2.08 ± 0.53	3.48 ± 0.59	4.23 ± 0.97	<0.001	<0.001	<0.001	<0.001

^a^Independent samples T-test was used to investigate differences between sexes for P_loss_. P_1_ = power in step 1; P_max_ = highest power production; P_loss_ = loss in power; P_mean_ = mean power.

Women demonstrated lower values for P_1_, P_max_, and P_mean_ and higher values for total duration (all p < 0.001). No sex difference was found for P_loss_ (p = 0.289). P_1_ did not differ between the different stair models (all p>0.05), whereas P_max_, P_mean_ and total duration were higher with increasing number of steps (all p<0.001, except for the difference in P_max_ and P_mean_ between 6 vs 12 step (p = 0.073 and p = 0.054)) (Table 2). A significant stair model-by-sex interaction effect was found for P_max_, indicating a lower difference between 3 vs 6 step (p = 0.003) and 3 vs 12 step (p<0.001) in women than in men. Likewise, the difference in P_mean_ between 3 vs 12 step (p<0.001) and 6 vs 12 step (p = 0.019) was lower in women than in men. Pearson’s r for power variables varied between 0.71 and 0.83 for P_1_ and between 0.82 and 0.95 for P_max_ and P_mean_, which showed moderate to very strong correlations between the different stair models (3-6-12 step) (Table 3).

**Table 3:** Pearson’s r correlation coefficients between the different stair models (3-6-12 steps) for men (♂, n = 59) and women (♀, n = 57).

	Pearson’s r*					
	3–6 step stair		3–12 step stair		6–12 step stair	
	♂	♀	♂	♀	♂	♀
P_1_ (W/kg)	0.737	0.779	0.738	0.833	0.706	0.823
P_max_ (W/kg)	0.880	0.874	0.820	0.887	0.862	0.927
P_mean_ (W/kg)	0.906	0.884	0.851	0.897	0.894	0.952
Total duration (s)	0.654	0.856	0.685	0.867	0.930	0.937

*Pearson’s r were all significant at p<0.001. P_1_ = power in step 1; P_max_ = highest power production; P_mean_ = mean power.

Within the different stair models, P_1_ and P_max_ showed moderate to strong relationships (r between 0.64 and 0.78, all p <0.001). In the 12-step stair model, P_loss_ was significantly related to P_1_ in both sexes (r = -0.27 in women and r = -0.45 in men; p <0.05) and to P_max_ in men only (r = -0.45, p <0.001).

### Test-retest reliability

Mean differences for SC parameters between test and retest session are displayed in Table 4. P_1_, P_max_ and P_mean_ showed good to excellent relative reliability in all staircases, with ICC’s varying between 0.79 and 0.95 (only P_1_ of the 12-step stair showed moderate relative reliability with an ICC of 0.66). P_mean_ and P_max_ showed good absolute reliability, with CV’s ranging between 6.63–9.85% and MDC’s ranging between 2.30–3.53%. P_1_ showed high CV’s (12.61%, 14.51% and 17.72% for the 3-, 6- and 12-step stair model, respectively) but low MDC’s (ranging between 5.33% and 7.83%). P_loss_ showed poor reliability (both absolute as relative, with ICC = 0.29, CV = -106.29% and MDC% = 29.01%). Total duration showed good reliability in the 6- and 12-step stair (CV = 5.38% and MDC% = 8.20% for the 6-step stair and CV = 4.49% and MDC% = 3.23% for the 12-step stair), but not in the 3-step stair model (CV = 12.60% and MDC% = 38.89%).

**Table 4:** Mean differences of stair-climbing performance parameters in the test-retest study sample (n = 53) and reliability coefficients. Mean differences were calculated as test–retest values.

	P_1_ (W/kg)	P_max_ (W/kg)	P_loss_ (%)	P_mean_ (W/kg)	Total duration (s)
**3-step stair**					
Mean difference ± SD	-0.19 ± 1.19	-0.29 ± 1.18	/	-0.24 ± 1.04	0.04 ± 0.16
CC (95% CI)	0.81 (0.69–0.88)	0.86 (0.77–0.92)	/	0.87 (0.79–0.92)	0.68 (0.50–0.80)
CV (%)	12.61	9.84	/	9.79	12.60
MDC%	5.33	3.13	/	3.53	38.89
**6-step stair**					
Mean difference ± SD	-0.24 ± 1.33	-0.37 ± 1.33	/	-0.20 ± 1.01	0.01 ± 0.14
ICC (95% CI)	0.79 (0.66–0.87)	0.89 (0.81–0.93)	/	0.90 (0.83–0.94)	0.94 (0.89–0.96)
CV (%)	14.51	9.85	/	9.00	5.38
MDC%	6.28	2.88	/	3.19	8.20
**12-step stair** ^a^					
Mean difference ± SD	-0.26 ± 1.60	-0.20 ± 1.20	-1.17 ± 14.40	-0.32 ± 0.75	0.07 ± 0.18
ICC (95% CI)	0.66 (0.48–0.79)	0.92 (0.86–0.95)	0.29 (0.01–0.52)	0.95 (0.91–0.97)	0.97 (0.95–0.98)
CV (%)	17.72	8.63	-106.29	6.63	4.49
MDC%	7.83	2.47	29.01	2.30	3.23

ICC = intraclass correlation coefficient; CI = confidence interval; CV = coefficient of variation; MDC = minimal detectable change; P_1_ = power in step 1; P_max_ = highest power production; P_loss_ = loss in power; P_mean_ = mean power.

^a^n = 50. Data of 3 subjects in the retest were missing because of issues in the manufacturer’s software.

## Discussion

This study investigated SC power on different stair models using a body-fixed sensor and showed that: 1) SC performance can be evaluated by means of three different parameters: the initial power on the first step or P_1_, the maximal power reached or P_max_, and the maintenance of power near the end of the stairs or P_loss_ (only measurable on a regular flight of stairs); 2) P_1_ and P_max_ showed moderate to strong relationships within a 3-, 6- and 12-step staircase, while P_loss_ was weakly related to P_1_ and P_max_; 3) P_1_ and P_max_ showed good reliability, whereas P_loss_ showed poor reliability in our study sample of community-dwelling older adults.

Measuring lower-limb muscle power often involves isolated joint movements, which do not capture the complexity of daily-life movements [11]. For implementing lower-limb muscle power measurements in daily-life practice, tests should be functional, easy and low-cost [13]. Timed stair tests are frequently used in clinical practice [25]. Based on timed SC, a mean power value can be estimated [14, 19]. However, SC is in reality a complex and dynamic activity of daily living which requires strength, coordination and balance [17]. A lot of information is missing when estimating just one mean power value. When looking into the kinematics of SC, ascending a step involves two phases [15]: 1) a rise phase, where there is vertical velocity of the body; and 2) a stance phase, where there is no vertical velocity. Power is only produced in the rise phase of the step.

Accelerometers or body-fixed sensors allow us to analyze the SC movement in detail, as they can provide information about every single phase of every single step [15]. SC was previously measured with body-fixed sensors [15, 26]. However, no study has investigated the evolution of SC power production per step. By plotting the mean power production from the first step to the last, we were able to differentiate between different phases in the SC performance. First, there is an acceleration phase or a phase where power is building up from zero in the first step, here defined as P_1_. Second, there is a phase where the maximal performance is reached or P_max_. And third, in a regular staircase with a sufficient amount of steps, we see a fatigue-related drop in power or P_loss_. This subdivision in three phases is in line with neuromuscular function assessments in isometric or dynamic tests, i.e. 1) rate of force/power development in the initial phase [27, 28], 2) peak force/power production in the phase of maximal performance [28] and 3) a fatigue-related reduction in force/power [29].

As participants may change their movement pattern or speed between separate test sessions, test-retest reliability of SC performance parameters should be evaluated [15]. We have previously reported excellent test-retest results for duration and P_max_ on a 6-step stair model (ICC = 0.93–0.94 and CV = 4.0–6.3%) [15]. These results are in line with the current study, where we found good to excellent reliability for total duration, P_mean_ and P_max_ on the 6- and 12-step stair model. Even though P_mean_ and P_max_ also showed good reliability in the 3-step stair model, total duration was less reliable. This indicates that caution is advised when total duration on a short flight of stairs is used to estimate mean power values. In addition, P_1_ appeared less reliable than P_max_ or P_mean_, which is in line with previous reports on rate of force/power development, indicating that early phases of force/power production are more prone to variability [28, 30]. Furthermore, P_loss_ showed poor reliability (ICC = 0.29, CV = -106.29%, MDC% = 29.01%) in our study sample and should not be used.

As most clinical practices do not have access to a full flight of stairs, the possibility to measure SC parameters on a smaller, and even transportable, staircase is valuable. Results showed that P_1_ did not differ between a 3-, 6- and 12-step stair model. This indicates that subjects started the SC test in a similar way on the different staircases, regardless of the number of steps. Absolute values of P_max_ were higher with increasing number of steps (P_max_ 3<6<12 step stair), indicating that 3 to 6 steps are insufficient to reach the real P_max_. However, a high correlation was found between the P_max_ values of the three different staircases (Pearson’s r ranged between 0.82 and 0.93), indicating that the values on a smaller staircase are a good estimate of the P_max_ reached on the 12-step stair. Similar values of MDC% values were found for the different parameters between the different staircases, supporting the idea that staircase models with a smaller number of steps can provide similar information as a regular staircase. As absolute values do not match between the different stair models, follow-up measurements of an individual should always be performed on the same stair model.

This study has some limitations. We have included a small sample of rather well-functioning older adults. As power is declining from middle-age onwards [5], the role of P_1_, P_max_ and P_loss_ should be examined further in a more diverse sample, including middle-aged adults and mobility-limited elderly. Assessing SC can be challenging in mobility-limited elderly and the risks of the test need to be balanced against the benefits. However, we propose the use of SC tests to detect early changes in muscle power prior to the initiation of mobility limitations, as it appears more sensitive to age-related declines than sit-to-stand tests [15].

## Conclusions

Three different performance phases can be distinguished during SC: an acceleration phase where power is built up, a phase of maximal performance and a phase of power loss. Measurements of SC power production with a single sensor on the lower back are found to be reliable across different staircase models. A small, transportable, 3-step staircase can be used for measuring power production in clinical practices with no access to regular staircases. However, absolute values are clearly lower in a 3-step stair model compared to a regular staircase, indicating that measurements to track performance changes over time should always be done using the same stair model. Future research should investigate the predictive value of SC power production on loss of independence and negative health outcomes with aging. In the meanwhile, a better insight in the trainability of the different components of power during SC (P_1_, P_max_ and P_loss_) can clarify the potential of SC in exercise programs.

## References

[1] Lexell J. Evidence for Nervous System Degeneration with Advancing Age. American Society for Nutritional Sciences. 1997;127:1011–3. doi: 10.1093/jn/127.5.1011S 9164286

[2] Gerstner GR, Giuliani HK, Mota JA, Ryan ED. Age-related reductions in muscle quality influence the relative differences in strength and power. Exp Gerontol. 2017;99:27–34. doi: 10.1016/j.exger.2017.09.009 28927901

[3] Alcazar J, Rodriguez-Lopez C, Delecluse C, Thomis M, Van Roie E. Ten-year longitudinal changes in muscle power, force, and velocity in young, middle-aged, and older adults. J Cachexia Sarcopenia Muscle. 2023. doi: 10.1002/jcsm.13184 36788413 PMC10067493

[4] Skelton DA, Greig CA, Davies JM, Young A. Strength, Power and Related Functional Ability of Healthy People Aged 65–89 Years. Age Ageing. 1994;23(5):371–7. doi: 10.1093/ageing/23.5.371 7825481

[5] Kostka T. Quadriceps maximal power and optimal shortening velocity in 335 men aged 23–88 years. Eur J Appl Physiol. 2005;95(2–3):140–5. doi: 10.1007/s00421-005-1390-8 16032419

[6] Van Roie E, Van Driessche S, Inglis AJ, Thomis M, Delecluse C. Rate of power development of the knee extensors across the adult life span: A cross-sectional study in 1387 Flemish Caucasians. Exp Gerontol. 2018;110:260–6. doi: 10.1016/j.exger.2018.06.021 29953950

[7] Foldvari M, Clark M, Laviolette LC, Bernstein MA, Kaliton D, Castaneda C, et al. Association of Muscle Power With Functional Status in Community-Dwelling Elderly Women. J Gerontol A Biol Sci Med Sci. 2000;55(4):M192–9. doi: 10.1093/gerona/55.4.m192 10811148

[8] Bean JF, Leveille SG, Kiely DK, Bandinelli S, Guralnik JM, Ferrucci L. A Comparison of Leg Power and Leg Strength Within the InCHIANTI Study: Which Influences Mobility More? J Gerontol A Biol Sci Med Sci. 2003;58A(8):728–33. doi: 10.1093/gerona/58.8.m728 12902531

[9] Metter EJ, Talbot LA, Schrager M, Conwit RA, Metter E, Jeffrey LA, et al. Arm-cranking muscle power and arm isometric muscle strengthare independent predictors of all-cause mortality in men. J Appl Physiol. 2004;96:814–21. doi: 10.1152/japplphysiol.00370.2003 14555682

[10] Alcazar J, Navarrete-Villanueva D, Manas A, Gómez-Cabello A, Pedrero-Chamizo R, Alegre LM, et al. “Fat but powerful” paradox: Association of muscle power and adiposity markers with all-cause mortality in older adults from the EXERNET multicentre study. Br J Sports Med. 2021 Nov 1;55(21):1204–11. doi: 10.1136/bjsports-2020-103720 33727213

[11] Alcazar J, Guadalupe-Grau A, García-García FJ, Ara I, Alegre LM. Skeletal Muscle Power Measurement in Older People: A Systematic Review of Testing Protocols and Adverse Events. Journals of Gerontology—Series A Biological Sciences and Medical Sciences. 2018;73(7):914–24. doi: 10.1093/gerona/glx216 29309534

[12] Zech A, Steib S, Freiberger E, Pfeifer K. Functional muscle power testing in young, middle-aged, and community-dwelling nonfrail and prefrail older adults. Arch Phys Med Rehabil. 2011;92(6):967–71. doi: 10.1016/j.apmr.2010.12.031 21514567

[13] Alcazar J, Losa-reyna J, Rodriguez-lopez C, Alfaro-acha A, Rodriguez-mañas L, Ara I, et al. The sit-to-stand muscle power test: An easy, inexpensive and portable procedure to assess muscle power in older people. Exp Gerontol. 2018;112:38–43. doi: 10.1016/j.exger.2018.08.006 30179662

[14] Bean JF, Kiely DK, LaRose S, Alian J, Frontera WR. Is Stair Climb Power a Clinically Relevant Measure of Leg Power Impairments in At-Risk Older Adults? Arch Phys Med Rehabil. 2007;88(5):604–9. doi: 10.1016/j.apmr.2007.02.004 17466729

[15] Van Roie E, Van Driessche S, Huijben B, Baggen R, Van Lummel RC, Delecluse C. A body-fixed-sensor-based analysis of stair ascent and sit-to-stand to detect age-related differences in leg-extensor power. PLoS One. 2019 Jan 1;14(1). doi: 10.1371/journal.pone.0210653 30653542 PMC6336282

[16] Hinman MR, O’Connell JK, Dorr M, Hardin R, Tumlinson AB, Varner B. Functional predictors of stair-climbing speed in older adults. Journal of Geriatric Physical Therapy. 2014;37(1):1–6. doi: 10.1519/JPT.0b013e318298969f 23835772

[17] Harper NG, Wilken JM, Neptune RR. Muscle function and coordination of stair ascent. J Biomech Eng. 2018 1;140(1). 10.1115/1.4037791 28857115

[18] Bean J, Ba SH, Mph DKK, Bs DC. Weighted Stair Climbing in Mobility-Limited Older People: A Pilot Study. J Am Geriatr Soc. 2002;50:663–70. doi: 10.1046/j.1532-5415.2002.50160.x 11982666

[19] Ni M, Brown LG, Lawler D, Bean JF. Reliability, Validity, and Minimal Detectable Change of Four-Step Stair Climb Power Test in Community- Dwelling Older Adults. Phys Ther. 2017;97(7):767–73. doi: 10.1093/ptj/pzx039 28444350 PMC6257032

[20] Psaltos DJ, Mamashli F, Adamusiak T, Demanuele C, Santamaria M, Czech MD. Wearable-Based Stair Climb Power Estimation and Activity Classification. Sensors. 2022;22(17). doi: 10.3390/s22176600 36081058 PMC9459813

[21] Hopkins WG, Marshall SW, Batterham AM, Hanin J. Progressive statistics for studies in sports medicine and exercise science. Med Sci Sports Exerc. 2009 Jan;41(1):3–13. doi: 10.1249/MSS.0b013e31818cb278 19092709

[22] Guralnik JM, Simonsick EM, Ferrucci L, Glynn RJ, Berkman LF, Blazer DG, et al. A short physical performance battery assessing lower extremity function: Association with self-reported disability and prediction of mortality and nursing home admission. Journals of Gerontology. 1994;49(2). 10.1093/geronj/49.2.m85 8126356

[23] Schober P, Schwarte LA. Correlation coefficients: Appropriate use and interpretation. Anesth Analg. 2018;126(5):1763–8. doi: 10.1213/ANE.0000000000002864 29481436

[24] Koo TK, Li MY. A Guideline of Selecting and Reporting Intraclass Correlation Coefficients for Reliability Research. J Chiropr Med. 2016;15(2):155–63. doi: 10.1016/j.jcm.2016.02.012 27330520 PMC4913118

[25] Nightingale EJ, Pourkazemi F, Hiller CE. Systematic review of timed stair tests. J Rehabil Res Dev. 2014;51(3):335–50. doi: 10.1682/JRRD.2013.06.0148 25019658

[26] Verlaan L, Storken G, Ic H, Grimm B. Accelerometer Based Stair Climbing in Healthy Subjects: Reference Data and Demographic Differences. 2019;9(5):7–12.

[27] Maffiuletti NA, Aagaard P, Blazevich AJ, Folland J, Tillin N, Duchateau J. Rate of force development: physiological and methodological considerations. European Journal of Applied Physiology. 2016; 116(6):1091–116. doi: 10.1007/s00421-016-3346-6 26941023 PMC4875063

[28] Van Driessche S, Van Roie E, Vanwanseele B, Delecluse C. Test-retest reliability of knee extensor rate of velocity and power development in older adults using the isotonic mode on a Biodex System 3 dynamometer. PLoS One. 2018;13(5):1–12. doi: 10.1371/journal.pone.0196838 29723252 PMC5933798

[29] Dobbeleer DL, Beckwée D, Arnold P, Baudry S, Beyer I, Demarteau J, et al. Comparison between two different handgrip systems and protocols on force reduction in handgrip assessment. Gerontology. 2023. doi: 10.1159/000530227 37276855

[30] Buckthorpe MW, Hannah R, Pain TG, Folland JP. Reliability of neuromuscular measurements during explosive isometric contractions, with special reference to electromyography normalization techniques. Muscle Nerve. 2012 Oct;46(4):566–76. doi: 10.1002/mus.23322 22987699

